# Comparison of APACHE II and SAPS II Scoring Systems in Prediction of Critically Ill Patients’ Outcome 

**Published:** 2017-01-08

**Authors:** Hamed Aminiahidashti, Farzad Bozorgi, Seyyed Hosein Montazer, Majid Baboli, Abolfazl Firouzian

**Affiliations:** 1 Department of Emergency Medicine, Faculty of Medicine, Mazandaran University of Medical Sciences, Sari, Iran.; 2 Department of Anesthesiology, Faculty of Medicine, Mazandaran University of Medical Sciences, Sari, Iran.

**Keywords:** APACHE, patient outcome assessment, critical illness, validation studies [publication type], emergency service, hospital

## Abstract

**Introduction:**

Using physiologic scoring systems for identifying high-risk patients for mortality has been considered recently. This study was designed to evaluate the values of Acute Physiology and Chronic Health Evaluation II (APACHE II) and Simplified Acute Physiologic Score (SAPS II) models in prediction of 1-month mortality of critically ill patients.

**Methods:**

The present prospective cross sectional study was performed on critically ill patients presented to emergency department during 6 months. Data required for calculation of the scores were gathered and performance of the models in prediction of 1-month mortality were assessed using STATA software 11.0.

**Results:**

82 critically ill patients with the mean age of 53.45 ± 20.37 years were included (65.9% male). Their mortality rate was 48%. Mean SAPS II (p < 0.0001) and APACHE II (p = 0.0007) scores were significantly higher in dead patients. Area under the ROC curve of SAPS II and APACHE II for prediction of mortality were 0.75 (95% CI: 0.64 - 0.86) and 0.72 (95% CI: 0.60 - 0.83), respectively (p = 0.24). The slope and intercept of SAPS II were 1.02 and 0.04, respectively. In addition, these values were 0.92 and 0.09 for APACHE II, respectively.

**Conclusion:**

The findings of the present study showed that APACHE II and SAPS II had similar value in predicting 1-month mortality of patients. Discriminatory powers of the mentioned models were acceptable but their calibration had some amount of lack of fit, which reveals that APACHE II and SAPS II are partially perfect.

## Introduction

Triage of high-risk patients in emergency department (ED) and focused and careful management of them might result in a drop in their mortality rate (1-4). A scoring model with high screening performance characteristics can provide considerable advantages for health systems. These advantages include prediction of patient outcome, evaluating the efficiency of treatments used, efficient pre- and in-hospital triage, and quality improvement of treatment measures and preventive plans (6). In addition, scoring systems are able to convert the severity of an illness into a number, which results in a common understanding between physicians for taking measures and developing quality control plans regarding patient care. Researchers have long attempted to design various scoring systems for this purpose. They have modified these systems to increase their efficiency, accuracy, and validity. Despite significant advances in these systems, unfortunately these models have had some deficiencies and limitations (5). These limitations include complicated calculations for some models, their high number of variables, and unevaluated validity in various clinical conditions. Therefore, research in this field is ongoing and new models are introduced each year.

Using physiologic scoring systems for identifying high-risk patients for death has been especially considered in recent years. To date, some physiologic scoring systems have been invented and introduced. One of the first physiologic scoring systems is Acute Physiology and Chronic Health Evaluation II (APACHE II), introduced by Knaus et al. in 1985. This model is calculated based on 12 physiologic criteria, age, and previous condition of the patient. Existing studies have revealed the close relation of this score with in-hospital and 1-month mortality in critically ill patients (7, 8). Simplified Acute Physiologic Score (SAPS II) is among other scoring models in this field, proposed by Le Gall et al. This model consists of 17 variables including 12 physiologic factors, age, type of admission, and 3 variables regarding underlying diseases (9). Predictive value of this model has been confirmed in different clinical conditions (10-12). These 2 models have been compared in different studies that have yielded somehow contradicting results (13-15). 

Therefore, the present study was designed aiming to evaluate and compare the values of APACHE II and SAPS II models in prediction of 1-month mortality of critically ill patients presented to emergency department (ED).

## Methods

**Study design and setting**

The present prospective cross sectional study was performed on critically ill patients admitted to Imam Khomeini Hospital, Sari, Iran, during February to June 2015 and assessed the accuracy of APACHE II and SAPS II in prediction of in hospital mortality. Ethics committee of Mazandaran University of Medical Sciences approved the protocol of the study. Informed consent was taken from patients. The researchers adhered to principles of Helsinki Deceleration. 

**Participants**

Critically ill patients were diagnosed based on appearance of patients, neurological assessment, respiratory status, cardiovascular assessment at time of admission to ED (Panel 1) (16) and were enrolled using convenience sampling. Participants lost to follow-up were excluded.

Age, gender, diagnosis impression, underlying diseases, vital signs, Glasgow Coma Scale (GCS), urinary output, need for ventilator, and length of intensive care unit (ICU) and hospital stay of all participants were gathered using a pre-designed checklist. Moreover, laboratory data including cell blood count (CBC), hematocrit, sodium, potassium, creatinine, bilirubin, and arterial blood gas analysis (pH, bicarbonate level, and oxygen and carbon dioxide pressure) were measured and recorded.

APACHE II and SAPS II scores were calculated during the first 24 hours after admission based on detailed method of calculations presented in previous studies (7, 17). 30-day mortality rate was assessed using patients’ medical records and calling them by the phone. Finally, patients were classified as alive and dead. 

**Statistical analysis**

The number of samples was calculated to be 82 patients based on a 50% prevalence of mortality in critically ill patients (18-20), considering a confidence interval (CI) of 95% (α=0.05), and a power of 80% (β=0.2). STATA software version 11.0 was used for data analysis. Qualitative variables are presented as frequency and percentage and quantitative factors are presented as mean and standard deviation. Mann-Whitney U test and Fisher's exact test were used for comparisons. Validations of the models were assessed using discriminatory power estimation, calibration of predictive models, or a combination of the two. The discriminatory power was evaluated through calculating area under the receiver operating characteristic (ROC) curve (AUC) with 95% CI. General calibration of the model was also evaluated through drawing a calibration plot. In this plot, the perfect calibration is the reference line with an intercept of zero and a slope of 1. The overall performance was eventually assessed via Brier score in order to evaluate predictive accuracy and reliability of the model. P value < 0.05 was considered statistically significant.

## Results

**Figure 1:**
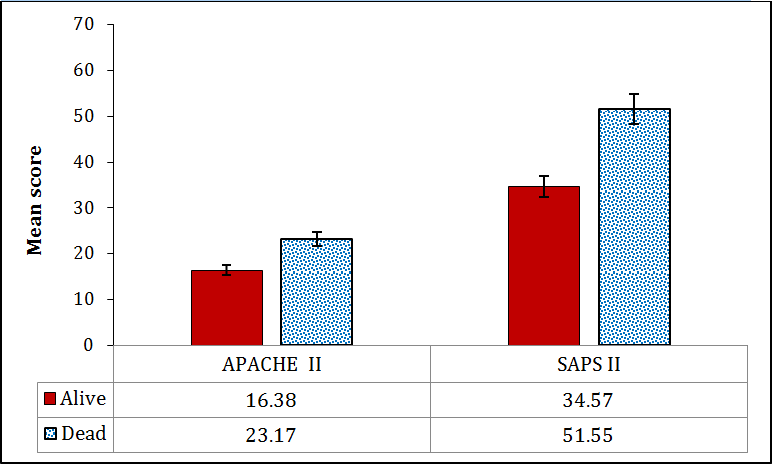
Mean score ± standard error of APACHE II and SAPS II in alive and dead patients (p < 0.001).

82 critically ill patients with the mean age of 53.45 ± 20.37 years were included (65.9% male). There were no cases of loss to follow-up. The most common cause of hospitalization was non-surgical (73.17%). Mean length of hospital stay was 4.78 ± 3.69 days and mortality rate was 48% (40 patients). Table 1 shows the baseline characteristics of patients. Age (p = 0.0006), reason of hospitalization (p = 0.002), and reason of ICU admission (p = 0.007) correlated with mortality. Mean SAPS II and APACHE II scores were 42.85 ± 19.67 and 19.69 ± 8.91, respectively. Mean SAPS II (p < 0.0001) and APACHE II (p = 0.0007) scores were significantly higher in dead patients (Figure 1). 

**Figure 2:**
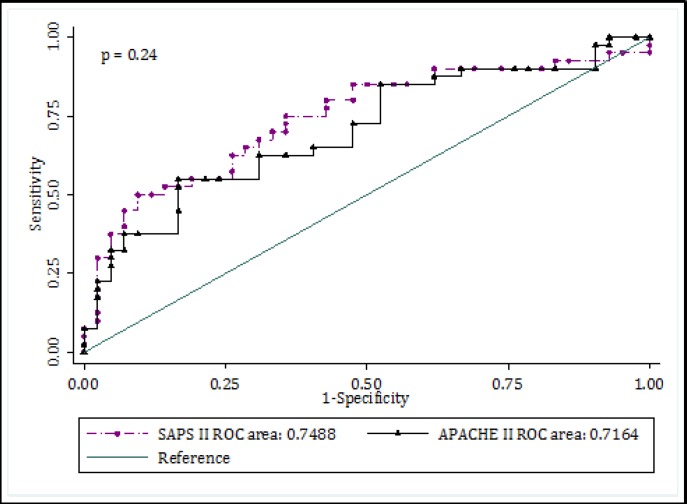
Receiver operating characteristic (ROC) curve of SAPS II and APACHE II in mortality prediction (p = 0.24

AUC of SAPS II and APACHE II for prediction of mortality were 0.75 (95% CI: 0.64 - 0.86) and 0.72 (95% CI: 0.60 - 0.83), respectively (p = 0.24) (Figure 2). 

**Figure 3:**
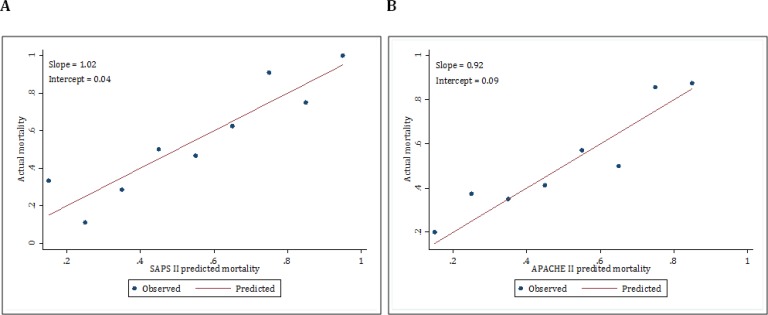
Calibration plots of SAPS II (A) and APACHE II (B

Calibration plots of these two scoring systems were presented in figure 3. The slope and intercept of SAPS II were 1.02 and 0.04, respectively. In addition, these values were 0.92 and 0.09 for APACHE II, respectively. 

Overall performances of SAPS II and APACHE II are presented in table 2. Brier score of SAPS II and APACHE II were 0.201 and 0.213, respectively. In addition, reliability of 0.019 and 0.024 for SAPS II and APACHE II shows goodness of fit of them in prediction of mortality. 

**Panel 1:** Diagnostic criteria of critically ill patients

**Appearance**	**Neurological**	**Respiratory**	**Cardiovascular**
Gray skin Blue skin Mottled skin	Unresponsive Eyes open to pain only Fitting	Silent chest RR < 8 or > 30 Agonal respiration	PR < 50 PR > 150 SPB < 60

RR: respiratory rate per minute; PR: pulse rate per minute, SBP: systolic blood pressure (mmHg).

**Table 1:** Baseline characteristics of participants based on their outcome

**Factor**	**Alive**	**Death**	**Total**	**P value**
**Age (year)**	45.90 ± 20.78	61.38 ± 16.84	53.45 ± 20.38	0.0006
**Gender**				
Male	29 (69.05)	25 (62.50)	54 (65.58)	0.53
Female	13 (39.95)	15 (37.50)	28 (34.15)	
**Reason of hospitalization**				
Medical	24 (57.14)	36 (90.0)	60 (73.17)	0.002
Surgical (emergent)	13 (30.95)	4 (10.0)	17 (20.73)	
Surgical (Elective)	5 (11.90)	0 (0.0)	5 (5.10)	
**Underlying disease**				
None	31 (73.81)	17 (42.50)	48 (58.54)	0.06
Acute renal failure	0 (0.0)	1 (2.50)	1 (1.22)	
Carcinoma	2 (4.76)	8 (20.0)	10 (12.20)	
Metastasis	2 (4.76)	4 (10.0)	6 (7.32)	
Systemic weakness	3 (7.14)	5 (12.50)	8 (9.76)	
Other	4 (9.52)	5 (12.50)	9 (10.98	
**Reason of ICU admission**				
Cardiovascular	0 (0.0)	2 (5.0)	2 (2.44)	0.007
Infection	12 (28.57)	21 (52.50)	33 (40.24)	
Respiratory	5 (11.90)	7 (17.50)	12 (14.63)	
Neurologic	5 (11.90)	3 (7.50)	8 (9.76)	
Multiple trauma	10 (23.81)	2 (5.0)	12 (14.63)	
Head trauma	8 (19.05)	1 (2.50)	9 (10.98)	
Other	2 (4.76)	4 (10.0)	6 (7.32)	
**Length of ICU stay (day)**	3.38 ± 3.01	6.10 ± 3.83	4.77 ± 3.70	0.0003

Data are presented as mean ± standard deviation or number (%).

**Table 2:** Overall performances of SAPS II and APACHE II

**Model**	**Brier Score**	**Sanders resolution**	**Reliability**	**Goodness of fit (%)**
**SAPS II**	0.201	0.182	0.019	86.22
**APACHE II**	0.213	0.193	0.024	84.51

## Discussion

Results of the present study showed that APACHE II and SAPS II models have similar value in prediction of 1-month mortality of the patients. Calibration of the 2 models had some amount of lack of fit. The two models showed partial adherence to the reference line, which indicates that the models are partially perfect in prediction of mortality. Discriminatory power was acceptable for both models.

In comparison with the results of the present study, Alizadeh et al. have expressed that APACHE II has higher value in prediction of mortality and disability resulting from intoxication compared to SAPS II (13). Similar findings have been reported by Taghavi Gilani et al. (21). However, by paying close attention to the Taghavi Gilani et al. article, we can see that AUC is 0.83 for APACHE II model and 0.78 for SAPS II; this difference does not seem statistically different. Haddadi et al. also revealed the value of these models in patient mortality prediction (11). In contrast, Sungurtekin et al. showed higher value for SAPS II model compared to APACHE II (22). These differences might be due to variations in study population and sample size, duration of follow-up and participant selection criteria.

Although the discriminatory powers of both APACHE II and SAPS II models were in an acceptable range, findings show some amount of lack of fit. Therefore, calibration of the mentioned models is not completely perfect. In line with the present study, Beck et al. also displayed the external validation of the mentioned models with a similar pattern but its calibration was imperfect (23). In another study, Khwannimit and Greater also expressed that AUC for APACHE II model in prediction of critically ill patients’ mortality is 0.79, yet the calibration of this model is reported to be poor (24). This might be mainly due to disease etiology and data gathering method not being homogenous (9, 24). Recent studies have shown that data gathering errors have been common, especially regarding patients with high or low APACHE II and GCS scores, and this affects the predictive role of the mentioned models (25). However, in the present study we tried to minimize data gathering errors by training the resident before initiation of sampling.

Possibility of selection bias in this study should not be overlooked since the study was single centric and participant selection was done using convenience sampling. Other limitations of this study include etiology of participant admission not being homogenous. This affected model calibration and led to detection of some amount of lack of fit in the 2 studied models. 

## Conclusion:

The findings of the present study showed that APACHE II and SAPS II had similar value in predicting 1-month mortality of patients. Discriminatory powers of the mentioned models were acceptable but their calibration had some amount of lack of fit, which reveals that APACHE II and SAPS II are partially perfect.
